# Correction to “Global distribution of treatment resistance gene markers for leishmaniasis”

**DOI:** 10.1002/jcla.25117

**Published:** 2024-10-31

S. Salari, M. Bamorovat, I. Sharifi, and P. G. N. Almani, “Global Distribution of Treatment Resistance Genemarkers for Leishmaniasis,” *Journal of Clinical Laboratory Analysis* 36 (2022): e24599, https://doi.org/10.1002/jcla.24599.

In Samira Salari, Mehdi Bamorovat, Iraj Sharifi, Pooya Ghasemi Nejad Almani, “Global distribution of treatment resistance gene markers for leishmaniasis” (2022), Figure 2 was published without permission and has been removed. This does not affect the conclusions of the article.

We apologize for this error.

